# Molecularly Imprinted Polymer-Based Electrochemical Sensor for Rapid and Selective Detection of Hypoxanthine

**DOI:** 10.3390/bios12121157

**Published:** 2022-12-12

**Authors:** Diksha Garg, Neelam Verma, Monika

**Affiliations:** 1 Biosensor Technology Laboratory, Department of Biotechnology and Food Technology, Punjabi University, Patiala 147002, Punjab, India; 2 Department of Biotechnology, Mata Gujri College, Fatehgarh 140407, Punjab, India

**Keywords:** molecularly imprinted polymers, nanomaterials, hypoxanthine, food safety, meat freshness, electrochemical sensor

## Abstract

In this paper, we report on the coupling of an electrochemical transducer with a specifically designed biomimetic and synthetic polymeric layer that serves as a recognition surface that demonstrates the molecular memory necessary to facilitate the stable and selective identification of the meat-freshness indicator hypoxanthine. Consumer preferences and the food safety of meat products are largely influenced by their freshness, so it is crucial to monitor it so as to quickly identify when it deteriorates. The sensor consists of a glassy-carbon electrode, which can be regenerated in situ continuously, functionalized with molecularly imprinted polymers (MIPs) and a nanocomposite of curcumin-coated iron oxide magnetic nanospheres (C-IO-MNSs) and multiwalled carbon nanotubes (MWCNTs) that enhance the surface area as well as the electroactive characteristics. The electrochemical behavior of the fabricated sensor was analyzed by both cyclic voltammetry (CV) and electrochemical impedance spectroscopy (EIS). Differential pulse voltammetric studies revealed the rapid response of the proposed sol-gel-MIP/MWCNT/C-IO-MNS/GCE sensor to hypoxanthine in a concentration range of 2–50 µg/mL with a lower limit of detection at 0.165 μg/mL. Application of the newly fabricated sensor demonstrated acceptable recoveries and satisfactory accuracy when used to measure hypoxanthine in different meat samples.

## 1. Introduction

Meat consumption is rising among people and has become crucial to a balanced diet for humans since it is a great source of proteins and vitamins [1]. Meat quality, however, starts deteriorating immediately upon the death of the animal. Given the significance of quality and safety assurance, the public, government, and practitioners in the food business have all placed a lot of stress on this issue. Freshness is a crucial determinant of the quality of meat and meat products. It is among the most pertinent indexes utilized by the food industry and consumers to evaluate the nutritional content of meat products [2].

Simple, rapid, affordable, and reliable meat-quality monitoring systems are becoming a must in response to growing food safety concerns, particularly in developing nations [3]. Hypoxanthine is considered to be a potential biomarker for assessing meat quality and diagnosing various disorders since it is typically produced during the purine degradation process. Immediately after death, ATP (adenosine triphosphate) biosynthesis stops and starts degrading to IMP (inosine monophosphate), which contributes a pleasant, fresh flavor to meat. Further, it degrades to hypoxanthine and progressively increases in concentration over time, which imparts a bitter taste and an unpleasant smell, indicating an early stage of deterioration. Thus, the quantification of hypoxanthine reveals the quality status of meat and meat products [4]. At present, there are different approaches for detecting hypoxanthine, including capillary electrophoresis (CE), mass spectrometry, high-performance liquid chromatography (HPLC), spectrophotometry (MS), hydrophilic interaction liquid chromatography with UV-Vis spectrometry (HILIC-UV), electrochemical devices, and enzymatic biosensors [5,6,7]. Among them, enzyme-based biosensors have attracted attention, owing to their simplicity and robustness. However, enzyme denaturation caused by changes in the environment, laborious enzyme extraction procedures, and protease digestion are some of the weaknesses of enzymatic biosensors. The enzyme xanthine oxidase, employed in hypoxanthine biosensor development, is unable to discriminate between hypoxanthine and xanthine as both are intermediates of purine degradation as well as substrates for xanthine oxidase. Hypoxanthine acts as a precursor of xanthine and uric acid formation, indicating the complete spoilage of meat. [8]. To obviate this drawback of target specificity, we have evaluated the efficacy of employing electrochemical sensors with molecularly imprinted polymers (MIPs) to accomplish the quick and selective identification of hypoxanthine to predict the degradation status of meat.

MIPs are tailor-made synthetic polymers that imitate the functionality of naturally occurring ligand binding sites while having the minimal cost and heat insensitivity of natural systems. They have specific identifying sites that can recognize a specified analyte or group of analytes [9]. Imprinted polymers have a long history of use as reliable, durable, affordable, and adaptable materials for target-molecule binding. Solid-phase extraction, catalysis, drug delivery, and sensing are some of the applications of these cavities. MIP-recognition components have more recently been integrated with different signaling devices to generate target-specific sensors for a wide range of applications [10]. There are different ways to create and incorporate MIPs into sensors, such as that used by Hussain et al. (2019), to fabricate an electrochemical sensor for the detection of folic acid by the electropolymerization of pyrrole on a molybdenum carbide (Mo_2_C)-modified glassy-carbon electrode [11]. Our strategy makes use of the drop casting technique due to its superior adhesion qualities, high repeatability, ease of use, quick preparation time, and capacity to regulate film thickness and morphology. Unlike the classic MIP-based sensors that are generally single-use, as they involve multiple rinsing steps to elute the template from the cavities in the elution buffer, this approach enables the in situ regeneration of the sensors, eliminating the repeated washing steps, and allowing for the continuous detection of hypoxanthine.

Curcumin, the main bioactive compound of turmeric, is renowned for its great antioxidant, anticancer, and anti-inflammatory nature. Lungu et al. (2010) studied the electrochemical behavior of curcumin by cyclic and differential pulse voltammetry and found more stable and better-defined peak currents as compared to the bare glassy-carbon electrode (GCE) [12]. Herein, we propose the development of an MIP-based sensor for detecting hypoxanthine for the assessment of the freshness of meat. The curcumin-coated Fe_2_O_3_ nanospheres and cMWCNTs (carboxylated multi walled carbon nanotubes) were co-immobilized onto GCE to increase the surface area for MIP deposition and to limit selectivity loss due to the direct deposition of the MIPs onto the electrode. The MIPs were synthesized and drop-cast onto the nanomaterial-modified electrode. The fabricated sensor allowed for the continuous assessment of the meat samples by eliminating the repeated template elution steps.

## 2. Materials and Methods

### 2.1. Chemicals and Reagents

The substances 3-aminopropyltrimethoxysilane (APTMS) and tetraethyl orthosilicate (TEOS) and the multiwalled carbon nanotubes (MWCNTs) (95%, L: 20–30 nm, D: 0.5–2 nm, W: 1–2 nm) were purchased from Sigma-Aldrich (Darmstadt, Germany; www.sigmaaldrich.com). Hypoxanthine, xanthine, caffeine, ferric chloride hexahydrate (FeCl_3_ × 6H_2_O), hexadecyl trimethyl ammonium bromide (CTAB), ethylene glycol (EG), sodium chloride, potassium chloride, sodium dihydrogen phosphate, and potassium hydrogen phosphate were purchased from Himedia (Mumbai, Maharashtra, India, http://www.himedialabs.com). Potassium ferricyanide, potassium ferrocyanide, sodium acetate, ethanol, methanol, ammonium hydroxide solution, acetic acid, hydrochloric acid, sulfuric acid, and nitric acid were obtained from SDFCL (India, http://www.sdfine.com). All of the reagents were of an analytical grade and were utilized without any further processing. A CH Instruments electrochemical workstation (CHI660D) (CH Instruments, USA) was used for all electrochemical experiments.

### 2.2. Synthesis of Fe_2_O_3_ Magnetic Nanospheres

Synthesis of curcumin-functionalized iron oxide magnetic nanospheres (C-IO-MNSs) was achieved by co-precipitating Fe(II) and Fe(III) salts in an alkaline solution by following the procedure in the literature with minor modifications [13]. For this particular reaction, 0.5 M of FeCl_2_ and FeCl_3_ (1:2 ratio) was dissolved into water at 50 °C for 30 min under continuous stirring and a constant nitrogen flow. A quantity of 2.0 M ammonium hydroxide was added dropwise to the reaction mixture after the addition of 10% curcumin dissolved in dimethyl sulfoxide (DMSO). For around 45 min, the reaction was left to stir at 90 °C. The precipitates were periodically neutralized by washing them with distilled water and then with ethanol. After drying in a hot-air oven at 60 °C, the precipitates were used for characterization by FTIR and then stored for further use.

### 2.3. Carboxylation of MWCNTs

Carbon nanotubes must be functionalized and rendered soluble in physiological media so as to be used in bioanalytical and biological chemistry. Pure MWCNTs were carboxylated using a method described by [14] with little modification. Pristine MWCNTs were added to the HNO_3_:H_2_SO_4_ (1:3 ratio) mixture and refluxed at 90 °C for 2 h. The pH was then balanced by rinsing the pretreated MWCNTs with distilled water. The cMWCNTs were then vacuum-dried at 60 °C and preserved for future use.

### 2.4. Synthesis of Molecularly Imprinted Polymers

The MIPs were synthesized by sol-gel polymerization, according to earlier work performed by our research team, and the MIP has already been characterized. Specifically, 0.1 mM of hypoxanthine as a template, 0.4 mM APTMS as the functional monomer, and 2 mM TEOS as the cross-linker were dissolved in 25 mL of ethanol [15]. Then, 0.5 mL of conc. HCl was added to initiate the polymerization reaction, and the reaction mixture was kept under continuous stirring at 40 °C for 1 h. After that, the resultant solution was dried overnight at 100 °C and mechanically ground into a fine powder. Hypoxanthine was extracted from the dried polymer by washing it in an ethanol: ammonium hydroxide (7:3, *v*/*v*) solution until no hypoxanthine was detected via UV–Vis spectroscopy, and then it was allowed to dry overnight at 37 °C. A similar procedure was utilized to synthesize nonimprinted polymers (NIPs), except for the addition of the template hypoxanthine.

### 2.5. Fabrication of C-IO-MNSs/cMWCNTs/MIPs onto GCE

At first, to achieve a mirror-like, clean surface, a glassy-carbon electrode (GCE) was buffed with an alumina slurry of varying particle sizes (1.0, 0.3, and 0.05 m) on a pad. The GCE was then thoroughly rinsed several times using a mixture of DI water and ethanol (1:1), respectively. The drop-casting approach was used to fabricate conducting polymer-based working electrodes as reported earlier [16]. Secondly, 5 µL of the 1.0 mg/mL curcumin-coated Fe_2_O_3_ magnetic nanosphere solution was cast onto the cleaned surface of the GCE until it dried at ambient conditions. Then, the modified electrode was cleansed with DI water several times to dispose of the unbound particles and dried at room temperature. After that, 5 µL of MWCNT-COOH, followed by sol-gel MIPs, were drop-cast on the modified electrode, washed, and dried prior to use.

### 2.6. Characterization of Modified Electrodes

A CH Instruments electrochemical workstation (CHI660D) with a 3-electrode system was used to perform all of the electrochemical studies at room temperature. A 3 mm modified GCE, a platinum (Pt) wire, and saturated Ag/AgCl acted as the working electrode, counter electrode, and reference electrode, respectively. Morphological studies of the modified working electrode with C-IO magnetic nanospheres, MWCNTs, and MIPs were investigated by SEM (scanning electron microscopy) (JEOL Ltd., MA, USA).

By using electrochemical impedance spectroscopy (EIS) and cyclic and differential pulse voltammetry (DPV) techniques, the electrochemical characterization, along with the analytical efficiency of the modified electrode, was investigated. For CV, the experimental parameters were as follow: a potential range of 0.8 V to −0.3 V and a scan rate of 100 Mv/s. For EIS: an amplitude of 10 mV, a frequency range of 10 mHz to 100 kHz. For DPV: a potential range of −0.3 V to 0.6 V, a pulse width of 50 ms, a scan rate of 50 mV/s, a modulation amplitude of 50 mV, and a potential increment of 5 mV. All tests were conducted in a solution of 2.5 mM [Fe(CN)_6_]^3−/4−^ and 10 mM KCl in 10 mM PBS at room temperature.

### 2.7. Cross-Reactivity and Real Sample Application

To scrutinize the effect of potentially interfering compounds, 1 mg of interferent, such as xanthine or caffeine, was mixed into 25 mL of PBS and ethanol solution (9:1, *v*/*v*) for 10 min. Chicken, mutton, and pork were used as the meat samples. For each meat sample, a mortar and pestle were used to homogenize 5 g of the meat. After 15 min of stirring and incubation in 25 mL of the aforementioned buffer solution, the meat was centrifuged at 5000 rpm for 10 min. Hypoxanthine-eluted MIP electrodes were placed in 2 mL of the sample solution for the interferent and meat sample tests and then allowed to equilibrate for 4–5 min before the electrochemical analysis.

## 3. Results and Discussions

### 3.1. Fabrication and Characterization of C-IO-MNS/cMWCNT/sol-gel-MIP/GCE

**Figure 1:**
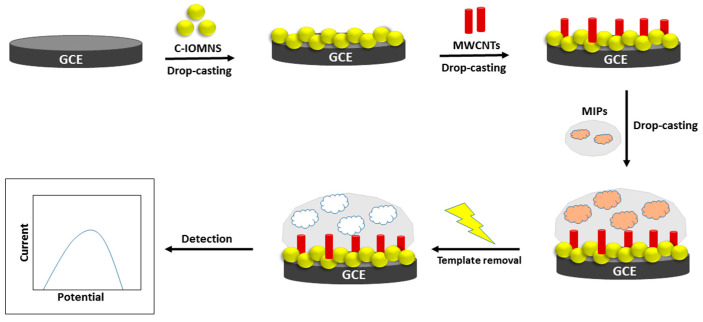
Schematic illustration of the MIP-sol-gel/MWCNT/C-IO-MNS/GC electrode fabrication. MIP: molecularly imprinted polymers, MWCNT: multi walled carbon nanotubes, C-IO-MNS: curcumin coated magnetic nanospheres, GC: glassy carbon electrode.

Detection of hypoxanthine with high selectivity and specificity succeeded through the careful fabrication of the nanomaterials and the MIP layer on the GCE. We utilized the nanocomposite layer of C-IO-MNSs and cMWCNTs sandwiched between the MIP layer and the GCE to enable rapid quantification (Figure 1). The C-IO-MNSs were synthesized using a green approach that could efficiently enhance electrode surface area, and the cMWCNTs could increase the electrical conductivity of immobilized material. MIPs are the receptors produced chemically by polymerizing the functional monomer along with a template molecule. Despite the fact that MIP technology has been put forth for diagnosis, sensing, and separation, it has not yet been proven to be effective for continuous sensing because traditional MIP sensors need to be washed repeatedly in order to regenerate. In this case, the functional monomer (APTMS) and cross-linker (TEOS) are embedded in the polymeric matrix on the GCE, and the target molecule initially forms a complex with them. Following the extraction of the target compound, binding sites that are compatible with the target analyte in terms of charge, shape, and size become visible. This results in the formation of molecular memories on the surface that permit the precise rebinding of the target. These methods for acquiring recognition sites feature binding properties that resemble those of antibody-antigen or enzyme-substrate systems. Additionally, this synthetic recognition method clearly outperforms actual enzymes in the following ways: They are suitable recognition components for sensors because they are innately stable, resilient, and simple to connect into an electronic device. This enables their deployment in a variety of applications, including any physiological fluids or at high temperatures.

**Figure 2:**
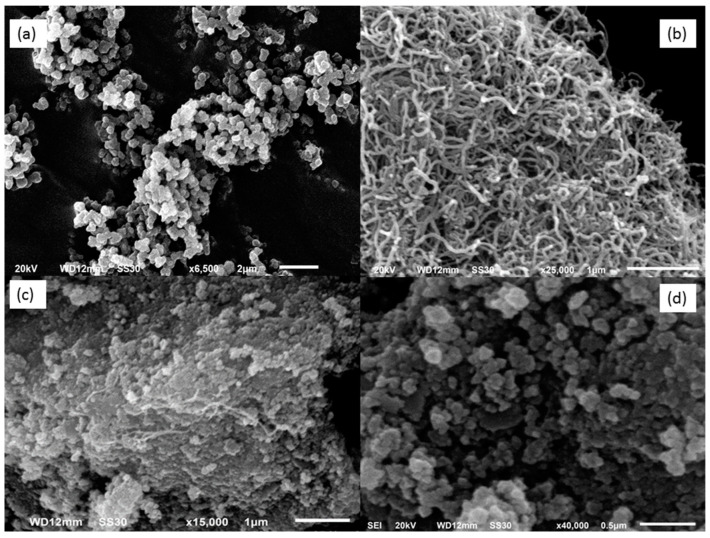
Surface morphology of the modified electrode with (**a**) curcumin-coated iron oxide magnetic nanospheres; (**b**) multiwalled carbon nanotubes; (**c**) sol-gel MIP before and (**d**) after template extraction by scanning electron microscopy (SEM).

The key to providing information about the surface of the changed electrodes at various phases is the morphological characteristics. As shown in Figure 2, the SEM was utilized to examine the surface morphology of the working electrode following the deposition of the C-IO-MNSs, MWCNTs, and the sol-gel-MIP films. The SEM of the C-IO-MNS-modified electrode is depicted in Figure 2a. The presence of uniform spherical structures confirmed the deposition of C-IO-MNSs. In Figure 2b, it can be seen that, after modification with MWCNTs, a uniform and homogenous three-dimensional cable-like structure develops on the surface of the C-IO-MNS-treated electrode. After the immobilization of the sol-gel MIPs, the electrode shows a very rough outer polymeric surface, bigger particles, and some degree of agglomeration, confirming the successful deposition of the surface-imprinted polymers (Figure 2c). After the removal of the template from the sol-gel-MIP/MWCNT/C-IO-MNS/GCE, the electrode shows the intermittent appearance of globular structures with gaps between them, verifying the cavity generation (Figure 2d).

**Figure 3:**
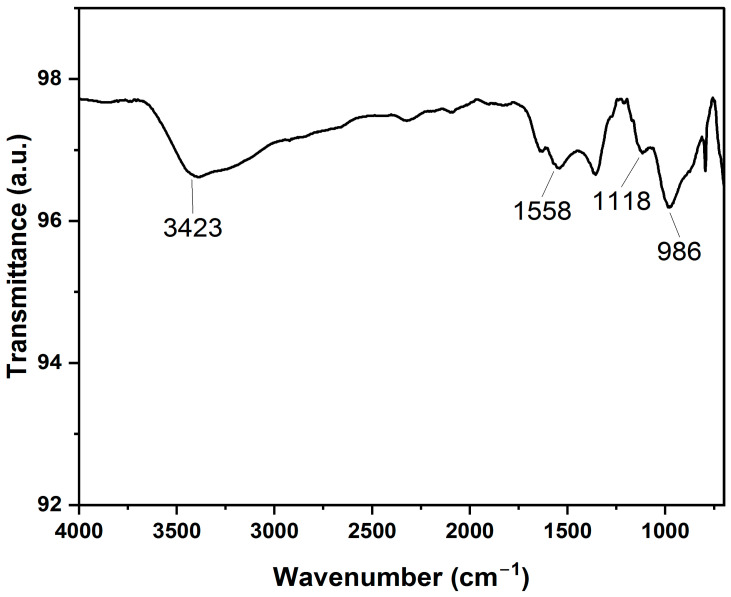
FTIR spectra of curcumin-coated iron oxide magnetic nanospheres (C-IO-MNSs).

A Fourier-transform infrared (FTIR) spectrometer was utilized to study the chemical properties of the curcumin-coated IO-MNSs. A dried powder made from the sample was placed on a KBr disk, and the spectrum was analyzed between 700 and 4000 cm^−1^ in an Impact-IO400 FTIR spectrometer, as shown in Figure 3. The FTIR analysis described the specific interaction of the IO magnetic nanospheres’ surfaces with the curcumin. To verify the interaction, the results were compared with those of previous studies and were found to be similar [13]. A sharp and broad spectrum around 3550–3100 cm^−1^ corresponds to the presence of -OH groups. A peak around 1558 cm^−1^ reveals the presence of the mixture of C=C and C=O groups, indicating the interaction between the curcumin and the nanospheres’ surfaces. A narrow peak at 1118 cm^−1^ corresponds to the stretching of the C-O-C group (C_6_H_5_-O-CH_3_ group). Moreover, in-plane bending of the -OH groups of enol moieties is indicated by the peak at 986 cm^−1^. Therefore, we can confirm the successful functionalization of the IO-MNSs with the curcumin.

### 3.2. Electrochemical Characterization of Fabricated C-IO-MNS/MWCNT/sol-gel-MIP electrode

**Figure 4:**
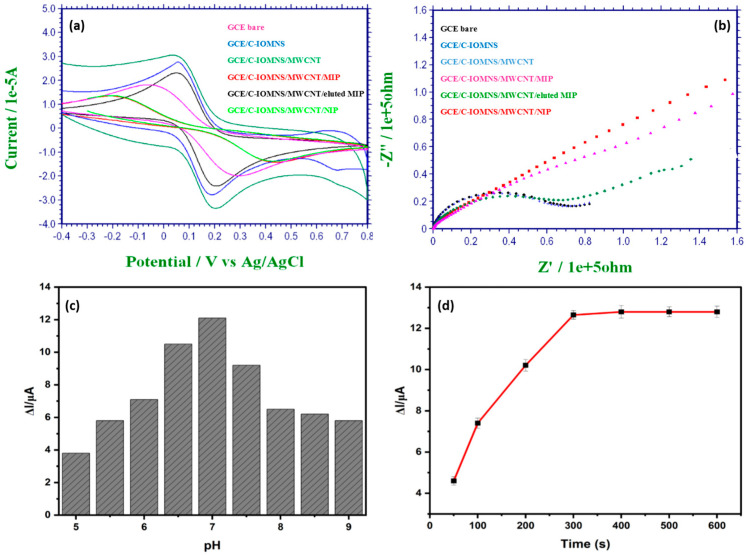
Electrochemical characterization by CV (**a**) and EIS (**b**) of bare GCE (A), C-IO-MNS/GCE (B), MWCNT/C-IO-MNS/GCE (C), sol-gel MIP/MWCNT/C-IO-MNS/GCE before (D) and after (E) template extraction, and NIP/MWCNT/C-IO MNS/GCE (F) recorded in K_3_[Fe(CN)_6_] (2.5 mM), containing 10 mM KCl and 10 mM PBS (pH = 7.0); impact of pH (**c**) and sample incubation time (**d**) on the functioning of the modified electrode. CV: cyclic voltammetry, EIS: electrochemical impedance spectroscopy, C-IO-MNS: curcumin coated magnetic nanospheres, MWCNT: multi walled carbon nanotubes, GCE: glassy carbon electrode, MIP: molecularly imprinted polymers, and NIP: non-imprinted polymers.

The electrochemical features of the modified electrode were examined by CV and EIS. The overlay of different CVs at each modification step is displayed in Figure 4a. For the preparation of the C-IO-MNS/cMWCNT/sol-gel-MIP/GCE sensor, redox-active materials, such as curcumin-coated iron oxide magnetic nanospheres (C-IO-MNSs) and multiwalled carbon nanotubes (MWCNTs) were deposited between the glassy-carbon electrode and the MIPs [16]. Following the modification with C-IO-MNSs, the redox peak current increased (from 1.819 × 10^−5^ A to 2.787 × 10^−5^ A). Additionally, when the electrode’s surface was functionalized with the cMWCNTs, the redox peak increased even further to 3.062 × 10^−5^ A. These findings suggest that C-IO-MNSs and cMWCNTs might enhance the electrocatalytic effect, as well as the electrochemical signal response. By comparing the acquired CVs for the sol–gel-MIP/cMWCNT/C-IO-MNS/GCE before (1.362 × 10^−5^ A) and after (2.306 × 10^−5^ A) removing the hypoxanthine, it can be seen that the removal of the template from the polymer significantly increases the currents of the redox couple. This occurs most likely because the cavities created by the extraction of the hypoxanthine from the polymeric network allow the reagent to scan more effectively toward the surface of the electrode. Precisely because the C-IO-MNSs’ and cMWCNTs’ deposition enhanced the electrochemical properties, the redox current peaks of the MIP after template extraction were much higher than that of the bare electrode. The CV curve of the NIP/cMWCNT/C-IO-MNS/GC electrodes resembled the MIP curve prior to elution (1.353 × 10^−5^ A).

EIS is a useful electrochemical approach for studying the interfacial features of fabricated electrodes. The curve obtained in the EIS characterization includes a semicircle and a line that correspond to the charge-transfer control and mass-transfer control processes, respectively. Ohmic resistance, which stands in for the solution resistance, is located where the first semicircle intersects the *X*-axis. Low charge-transfer resistances are revealed by a semicircle with a smaller diameter. Figure 4b depicts the Nyquist plots for the electrode at different modification stages. As can be seen, the charge-transfer resistance (RCT) value of the bare GCE dramatically decreases following GCE modification with the C-IO-MNSs and MWCNTs, showing that the C-IO-MNSs and MWCNTs both have good electroconductive capabilities and allow more electrons to transfer from the K_3_[Fe(CN)_6_] solution to the electrode surface than with a bare GCE. The surface area of the electrode and the synergistic interaction of the MWCNTs and the C-IO-MNSs both contribute to these improved performances. The charge-transfer resistance increased upon the deposition of the template-embedded MIP and NIP layer onto the C-IO-MNS/MWCNT/GCE, as a dense, nonconductive film has been created on electrode surface. After the template extraction, the charge-transfer resistance of the C-IO-MNS/MWCNT/MIP/GCE again decreases, which indicates that the conductivity of the electrode has increased due to cavity generation. According to the improved procedure, these impendence alterations show that the sol-gel-MIPs and nanomaterials altered the surface of the electrode.

### 3.3. Optimization of C-IO-MNS/cMWCNT/sol-gel-MIP/GCE sensor

To achieve the optimal sensor performance for rapid and accurate sample analysis, the impact of pH and sample incubation time were evaluated experimentally. The pH of the reaction mixture has an impact on the structure, as well as the operation, of the sol-gel-MIPs and the rate at which the electrons transfer. The sol–gel-MIPs could be destroyed by a test solution that is too basic or acidic. Sample solutions ([Fe(CN)_6_]^3−/4−^ of PBS including KCl) with different pH values ranging from 5 to 9 were used to analyze the impact of pH on the current response. The maximum response was attained at pH 7.0, as shown in Figure 3c. Therefore, 7.0 was chosen as the ideal pH for the sample solution. As displayed in Figure 4c, the height of the current peak increases moderately with the increase in the pH of the solution from 5 to 7, and then decreases from 7 to 9. Similarly, when the fabricated electrode is allowed to stand for template adsorption for 600 s, the current peak increases initially and then gradually stabilizes after 300 s, revealing the saturated adsorption of hypoxanthine (Figure 4d).

### 3.4. Detection of Hypoxanthine Using C-IO-MNS/cMWCNT/sol-gel-MIP/GCE

**Figure 5:**
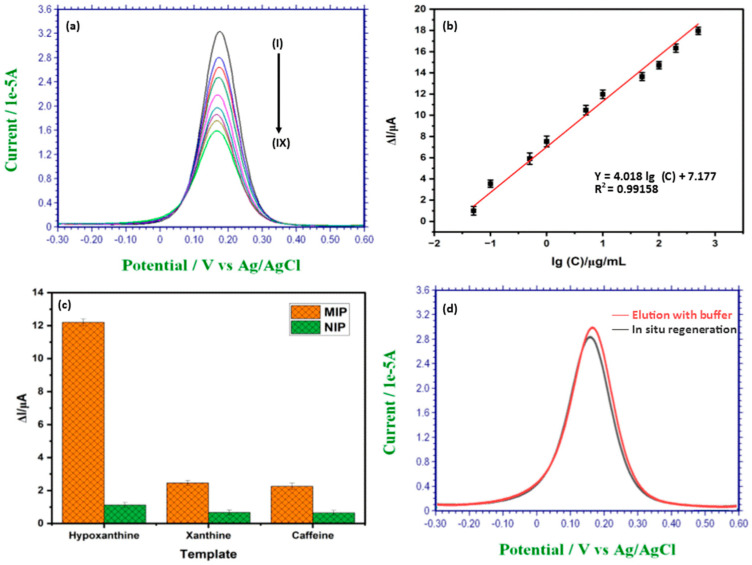
(**a**) DPV response of sol-gel MIP/MWCNT/C-IO-MNS/GCE (i) before (blank DPV) and (ii–ix) after addition of hypoxanthine in the concentration range of 2–50 µg/mL; (**b**) Corresponding calibration curve of the same hypoxanthine concentration range; (**c**) Selective recognition of hypoxanthine with the C-IO-MNS/MWCNT/sol-gel-MIP sensor among its analogues; (**d**) DPV voltammograms of modified electrode after elution with buffer and in situ regeneration. DPV: differential pulse voltammetry.

DPV was used to detect the concentration of hypoxanthine in the probe electrolyte that contained 10 mM phosphate buffered saline (PBS, pH 7.0) and 10 mM KCl. Under optimal conditions, the as-prepared C-IO-MNS/cMWCNT/MIP/GCE was kept in the hypoxanthine standard solutions, and the response was recorded at a potential range of −0.3 V to 0.6 V, a modulation amplitude of 50 mV, and a scan rate of 50 mV/s. Figure 5a depicts the obtained DPVs for the various hypoxanthine concentrations from 2 to 50 µg/mL. As seen in the figure, the current gradually decreased with the increase in hypoxanthine concentration, which indicated that more polymer sites became occupied by the compound, limiting electron transfer. The calibration curve (Figure 5b) showed that the change in the peak current (I) and the logarithmic hypoxanthine concentration (lgC) are in a linear relationship, with the regression equation being I (A) = 4.01 lgC + 7.17 (r^2^ = 0.9915) for the concentration between 2–50 µg/mL. The detection limit was established at 0.165 g/mL using the three values of the blank signals. The alteration of the IO-MNPs and the cMWCNTs, which may efficiently increase the surface area of the electrodes and raise the rate of electron transfer, may be the cause of the low detection limit. As a result, the sensor signal was amplified. The detection limit was then lowered, and the detecting sensitivity increased. In addition, this approach exhibited an extreme sensitivity towards hypoxanthine sensing along with a wider linear range that prevented sample serial dilution during actual detection. As demonstrated in Table 1, the approach to the C-IO-MNS/cMWCNT/sol-gel-MIP/GCE disclosed in the present study has a comparable concentration range and a lower detection limit with the previously fabricated electrochemical sensors for the detection of hypoxanthine. From the table, it is clear that our designed sensor has numerous advantages, such the lack of required purification steps, a lower cost, and a lower detection limit, over existing biosensors [7,17,18]. The electrode utilized in the sensor fabrication is relatively stable and can be reused for continuous monitoring. The key benefits of the currently developed sensor are its ease of manufacture, affordability, speed, ability to make precise detections, and high sensitivity.

### 3.5. Selectivity Study of the MIP Sensor

Selectivity in relation to other species is one of the characteristics of an ideal sensor. The selectivity of the sol-gel molecularly imprinted electrode originates from its stability. Selectivity is greatly influenced by two important variables: functional groups and molecule size. As a result, substances with various sizes, functional groups, and structures exhibit low interference in MIP-based sensors. The electrochemical responses of MIP and NIP electrodes for hypoxanthine and other compounds were studied using the DPV approach to assess the selectivity of the produced sensor, and the relevant results are shown in Figure 5c. The imprinting factor (IF), which is the ratio of ΔIMIP to ΔINIP, was used to intuitively measure the selectivity of the interfering species. As shown in Figure 5c, hypoxanthine had an IF value of 10.89, whereas the other interfering species had IF values between 3.67 and 3.51. According to all of the findings, the suggested electrochemical sensor is more sensitive and selective to hypoxanthine molecules than to other interfering species. The variety of functional groups, size, and conformation of the binding cavities that match the hypoxanthine molecules in the MIP layer can be used to explain this. There was no substantial change in the current variation between the NIP- and MIP-modified electrode for interfering compounds. The ability of the MIP layers to selectively identify target analytes was excellent, and the selectivity of the proposed sensor was found to be sufficient.

### 3.6. Regeneration of the MIP Sensor

Regeneration of sensor is the major hurdle that limits the applicability of MIP-based sensing technologies for the continuous monitoring of analytes. In this study, we investigated the possibility of in the situ regeneration of a MIP-based sensor by using a controlled voltammetric technique to achieve the wash-free and repeatable use of MIP sensors. For the MIP regeneration, a fixed redox potential was applied to the electrode for the extraction of the template. Once the right redox voltage is provided to the electrode, the molecule that is particularly fixed in the receptor site of the MIP sensor can be oxidized and released because the oxidized product has poor binding affinity to the MIP receptor site, resulting in sensor regeneration. After the detection DPV scan, the sensor response to the background current can be reset with a 100% recovery ratio by applying a fixed redox potential of 0.9 V for 20 s. To validate the in situ regeneration technique, the MIP sensor was also regenerated with an elution buffer (ethanol: ammonium hydroxide, 7:3). As can be seen in Figure 5d, the in situ regenerated MIP sensor shows a comparable binding capacity or imprinting factor with the MIP sensor regenerated by utilizing a washing buffer.

### 3.7. Repeatability, Reproducibility, and Stability of MIP Sensor

**Figure 6:**
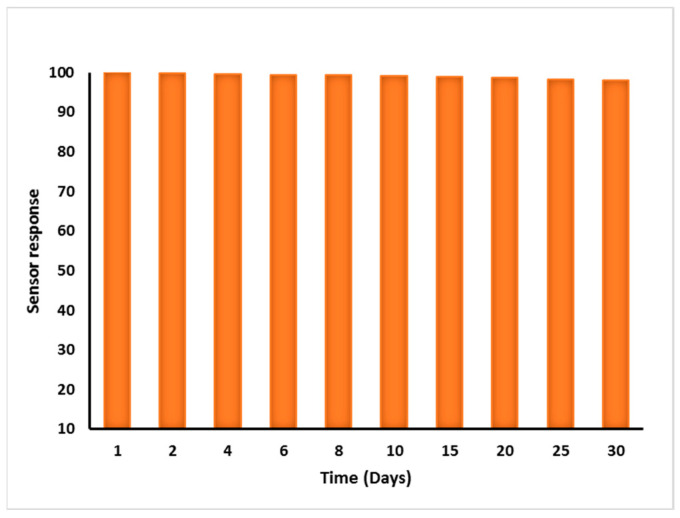
Storage stability of proposed C-IO-MNS/cMWCNT/sol–gel-MIP/GCE sensor.

For a device to be effective in real-world applications, repeatability, reproducibility, and stability are the critical factors of electrochemical sensors for freshness estimations. The C-IO-MNSs, cMWCNTs, and MIPs were deposited on the GCE surface. The sensor could be readily used and repeatedly regenerated in situ by supplying constant potential to the imprinted working electrode without any washing procedures. The potential repels the bound hypoxanthine molecules from the sol-gel MIP layer, resulting in prolonged reusability. The proposed sensor exhibited a stable current response when used 10 times after in situ regeneration; slight output changes were witnessed throughout a period of 30 days of storage at room temperature (Figure 6). In addition, the sensor displayed no considerable relative signal shift while being used regularly for 5 days, revealing high reproducibility and reliability.

### 3.8. Application of MIP Sensor for Hypoxanthine Analysis in Meat Samples

To evaluate the performance of the C-IO-MNS/cMWCNT/sol-gel-MIP/GCE sensor in real samples, chicken, pork, and goat meat samples were analyzed. The extraction of hypoxanthine from different meat samples was carried out using a simple method proposed by [4]. The meat samples were spiked with a known amount of hypoxanthine and the percentage recovery was determined by the developed sensor. All of the samples were analyzed in triplicate. The recoveries of exogenously added 5 µg/mL and 10 µg/mL hypoxanthine are shown in Table 2. This shows that the new sensor has high accuracy and great potential for real sample analysis. According to the real sample analysis results, it can be concluded that the proposed C-IO-MNS/MWCNT/sol-gel MIP/GCE has high potential for the estimation of hypoxanthine amounts in biological samples with satisfactory accuracy and precision.

## 4. Conclusions

Here, we report on the application of a MIP-based electrochemical sensor for achieving hypoxanthine detection in meat products. The sensor consists of curcumin-coated iron oxide magnetic nanospheres and multiwalled carbon nanotubes as redox-active nanomaterials, along with a target-specific antibody, such as MIPs. Under optimal working conditions, the proposed modified electrode exhibited a linear range of 2–50 µg/mL, a low detection limit of 0.165 µg/mL, and it selectively distinguished hypoxanthine from among structural analogs. In addition, the MIP-functionalized electrode can be regenerated in situ and utilized for continuous analysis without time-consuming washing steps, thus offering significant benefits over conventional detection techniques. The application of the newly fabricated sensor demonstrated acceptable recoveries and satisfactory accuracy when used to measure hypoxanthine in different meat samples.

## Figures and Tables

**Table 1:** Different hypoxanthine sensors developed to assess meat quality.

Working Electrode	Detection Method	LOD (µM)	Linear Range (µM)	Sample	Reference
XOD/HRP/µ-PAD	Colorimetric	13	36–293	Meat	[17]
XOD/NBT/Sol–gel/ CS	Colorimetric	4.1	4–35	Fish	[7]
XOD/U/PPy-pTS/Pt	Amperometric	5	5–500	Fish	[19]
XOD/CeNPs	Colorimetric	89	300–597	Fish	[18]
CR-RS films	Colorimetric	38.63	1–100	Chicken and Fish	[20]
MIP/ MWCNT/C-IO-MNS/GCE	DPV	1.21	15–367	Chicken, Pork, and Goat meat	This work

XOD: xanthine oxidase; HRP: horseradish peroxidase; NBT: nitro blue tetrazolium chloride; CS: chitosan; U: uricase, PPy-pTS: polypyrrole para-toluenesulfonate; LOD: limit of detection; Pt: platinum, CeNPs: cerium nanoparticles; CR-RS: curcumin/rice starch; DPV: differential pulse voltammetry; MIPs: molecularly imprinted polymers; MWCNTs: multiwalled carbon nanotubes; C-IO-MNSs: curcumin-coated iron oxide magnetic nanospheres; GCE: glassy-carbon electrode.

**Table 2:** Analytical recovery of spiked hypoxanthine in chicken, pork, and goat meat samples, as measured by sol–gel-MIP/MWCNT/C-IO-MNS/GCE based hypoxanthine sensor.

Meat Sample (n = 3)	Added Hypoxanthine (µg/mL)	Detected (µg/mL)	Recovery (%)	RSD (%)
Chicken	Unspiked	2.44 ± 0.05	-	2.0
	5	6.91 ± 0.13	92.8	1.9
	10	12.21 ± 0.21	98.2	1.7
Pork	Unspiked	6.67 ± 0.10		1.5
	5	11.43 ± 0.2	97.94	1.8
	10	16.50 ± 0.25	98.98	1.5
Goat meat	Unspiked	5.89 ± 0.10		1.7
	5	10.29 ± 0.19	94.49	1.9
	10	15.66 ± 0.25	98.57	1.6

## Data Availability

Not applicable.
